# NUScon: a community-driven platform for quantitative evaluation of nonuniform sampling in NMR

**DOI:** 10.5194/mr-2-843-2021

**Published:** 2021-11-25

**Authors:** Yulia Pustovalova, Frank Delaglio, D. Levi Craft, Haribabu Arthanari, Ad Bax, Martin Billeter, Mark J. Bostock, Hesam Dashti, D. Flemming Hansen, Sven G. Hyberts, Bruce A. Johnson, Krzysztof Kazimierczuk, Hengfa Lu, Mark Maciejewski, Tomas M. Miljenović, Mehdi Mobli, Daniel Nietlispach, Vladislav Orekhov, Robert Powers, Xiaobo Qu, Scott Anthony Robson, David Rovnyak, Gerhard Wagner, Jinfa Ying, Matthew Zambrello, Jeffrey C. Hoch, David L. Donoho, Adam D. Schuyler

**Affiliations:** 1 Department of Molecular Biology and Biophysics, UConn Health, Farmington, CT 06030, USA; 2 Institute for Bioscience and Biotechnology Research, National Institute of Standards and Technology and the University of Maryland, Rockville, MD 20850, USA; 3 Department of Cancer Biology, Dana Farber Cancer Institute, Boston, MA 02215, USA; 4 Department of Biological Chemistry and Molecular Pharmacology, Harvard Medical School, Boston, MA 02115, USA; 5 Laboratory of Chemical Physics, NIDDK, National Institutes of Health, Bethesda, MD 20892, USA; 6 Department of Chemistry and Molecular Biology, University of Gothenburg, Box 465, Gothenburg 405 30, Sweden; 7 Department of Biochemistry, University of Cambridge, Cambridge CB2 1GA, UK; 8 Division of Preventive Medicine, Brigham and Women's Hospital, Harvard Medical School, Boston, MA 02215, USA; 9 Department of Structural and Molecular Biology, Division of Biosciences, University College London, London WC1E 6BT, UK; 10 Structural Biology Initiative, CUNY Advanced Science Research Center, New York, NY 10031, USA; 11 Centre of New Technologies, University of Warsaw, 02-097 Warsaw, Poland; 12 Department of Electronic Science, Fujian Provincial Key Laboratory of Plasma and Magnetic Resonance, Xiamen University, Xiamen 361005, China; 13 Centre for Advanced Imaging, The University of Queensland, 4072 St Lucia, Queensland, Australia; 14 Department of Chemistry and Nebraska Center for Integrated Biomolecular Communication, University of Nebraska-Lincoln, Lincoln, NE 68588-0304, USA; 15 Department of Electronic Science, Biomedical Intelligent Cloud R&D Center, Fujian Provincial Key Laboratory of Plasma and Magnetic Resonance, National Institute for Data Science in Health and Medicine, Xiamen University, Xiamen 361005, China; 16 Department of Molecular and Cellular Biochemistry, Indiana University, Bloomington, IN 47405, USA; 17 Department of Chemistry, Bucknell University, Lewisburg, PA 17837, USA; 18 Department of Statistics, Stanford University, Stanford, CA 94305, USA

## Abstract

Although the concepts of nonuniform sampling (NUS​​​​​​​) and non-Fourier spectral reconstruction in multidimensional NMR began to emerge 4 decades ago [Bibr bib1.bibx4], it is only relatively recently that NUS has become more commonplace. Advantages of NUS include the ability to tailor experiments to reduce data collection time and to improve spectral quality, whether through detection of closely spaced peaks (i.e., “resolution”) or peaks of weak intensity (i.e., “sensitivity”). Wider adoption of these methods is the result of improvements in computational performance, a growing abundance and flexibility of software, support from NMR spectrometer vendors, and the increased data sampling demands imposed by higher magnetic fields. However, the identification of best practices still remains a significant and unmet challenge. Unlike the discrete Fourier transform, non-Fourier methods used to reconstruct spectra from NUS data are nonlinear, depend on the complexity and nature of the signals, and lack quantitative or formal theory describing their performance. Seemingly subtle algorithmic differences may lead to significant variabilities in spectral qualities and artifacts. A community-based critical assessment of NUS challenge problems has been initiated, called the “Nonuniform Sampling Contest” (NUScon), with the objective of determining best practices for processing and analyzing NUS experiments. We address this objective by constructing challenges from NMR experiments that we inject with synthetic signals, and we process these challenges using workflows submitted by the community. In the initial rounds of NUScon our aim is to establish objective criteria for evaluating the quality of spectral reconstructions. We present here a software package for performing the quantitative analyses, and we present the results from the first two rounds of NUScon. We discuss the challenges that remain and present a roadmap for continued community-driven development with the ultimate aim of providing best practices in this rapidly evolving field. The NUScon software package and all data from evaluating the challenge problems are hosted on the NMRbox platform.

## 1 Introduction

[Bibr bib1.bibx21] devised the conceptual basis for converting multiple-resonance NMR experiments into multidimensional experiments by parametric sampling of the free induction decays (FIDs) along “indirect time dimensions.” The subsequent application of the discrete Fourier transform (DFT) to the analysis of pulsed NMR experiments revolutionized NMR spectroscopy, opened the door to multidimensional experiments, and resulted in the 1991 Nobel Prize in Chemistry being awarded to Richard Ernst [Bibr bib1.bibx15]. However, despite the resulting advances in the development and application of NMR to challenging problems in the biomedical sciences, the limitations of the DFT also became apparent. The DFT operates on a series of equally spaced values measured over time and identifies the weighted sum of frequency components (i.e., the Fourier basis terms) needed to represent the time series. The requirement that the input data be equally spaced results in on-grid uniform sampling (US). The constraints of US are demonstrated by the following three observations: (1) sampling must be performed to an evolution time of $\pi \times T_{2}$ in order to resolve a pair of signals separated by one linewidth; (2) uniform data collection beyond $1.26\times T_{2}$ reduces the signal-to-noise ratio (SNR) [Bibr bib1.bibx30], a proxy for sensitivity, with the majority of SNR obtained by $∼0.6\times T_{2}$ ; and (3) sampling must be performed rapidly to avoid signal aliasing described by the Nyquist sampling theorem [Bibr bib1.bibx38], which means that attaining high resolution along any indirect dimension that is sampled parametrically will be costly in terms of the data acquisition time. These simple observations result in a forced compromise between sensitivity, resolution, and experiment time. These DFT limitations are exacerbated at higher magnetic fields, where increased dispersion requires shorter sampling intervals, resulting in the acquisition of more samples in order to reach the same evolution time as the corresponding experiment collected at lower fields. Higher-dimensionality experiments also help access the increased resolution afforded by high-field magnets by introducing separation of closely spaced peaks along perpendicular dimensions but at the cost of longer experiment times.

As a consequence of the fundamental limitations of US, there has been an ongoing effort to develop nonuniform sampling (NUS) schemes and non-Fourier spectral reconstruction methods in multidimensional NMR. NUS allows for a subset of the FIDs from the US grid to be collected. NUS spectral-processing methods reconstruct the missing data instead of replacing them with zeros, which is the inherent consequence of applying the DFT to NUS data. To date, the field has developed numerous spectral reconstruction methods and novel FID sampling strategies (e.g., Poisson gap sampling, [Bibr bib1.bibx18]; quantile sampling, [Bibr bib1.bibx9]), yet quantitative tools needed for the robust analysis of these methods and the development of standards have been comparatively slow to emerge. The field of NUS has often relied upon heuristics to design experiments (e.g., selecting sampling coverage or setting adjustable parameters for reconstruction algorithms). There are numerous reports based on a single experiment, a single sample schedule, or select 1D traces through “representative peaks” – all of which are prone to over-interpretation. While these investigations have revealed important aspects of NUS, they often carry limited applicability to other experiments. In addition, the nonlinear nature of non-Fourier methods and the wide range of data characteristics (e.g., sparsity of signals in the spectrum, overlapping peak positions, and dynamic range of peak intensities) makes the identification of best practices both a challenge and an imperative. Extensive reviews have been undertaken ([Bibr bib1.bibx3]; [Bibr bib1.bibx32]; [Bibr bib1.bibx5]; [Bibr bib1.bibx42]), but without a common set of problems or community-adopted metrics, best practices remain elusive.

The field of compressed sensing CS has pursued intense theoretical investigations, revealing, among other insights, the importance of incoherence in the sampling scheme and the phenomenon of phase transitions in the ability to recover the spectrum as a function of the number of samples collected and the number of non-zero elements in the spectrum [Bibr bib1.bibx11]. However, the theoretical work in CS assumes a measure of sparsity as defined by a strict counting of non-zero values in the spectrum. This condition is violated in NMR by experimental noise and by the broadness of peaks from decaying signals, as compared to delta spikes. In addition, CS theory is founded on random sampling, whereas NUS often employs biased sampling. CS theory offers significant qualitative insights for NMR [Bibr bib1.bibx7], but because its quantitative predictions are not directly applicable to NMR, additional research on CS-based NUS in NMR is needed.

The design of optimal sampling schemes for multidimensional NMR is still an open question. Further, an explosion of computational approaches for spectral estimation from NUS data poses the additional challenge of identifying optimal spectral reconstruction methods and parameter choices. The approaches vary widely and can be loosely categorized by the extent to which the algorithms impose assumptions about the nature of the signals. At one extreme are the parametric methods, which explicitly model the signals: Bayesian [Bibr bib1.bibx55], maximum likelihood [Bibr bib1.bibx8], SMILE [Bibr bib1.bibx54], and CRAFT [Bibr bib1.bibx25]. At the other extreme are the non-parametric methods, which make no assumptions about the nature of the signals but typically assume noise is randomly distributed: maximum entropy [Bibr bib1.bibx48], NESTA [Bibr bib1.bibx51], and CS. Methods like multi-way decomposition [Bibr bib1.bibx40], which describe the recovered spectra as an outer product of 1D projections, fall in between the parametric and non-parametric. Regardless of the approach, a number of factors complicate the critical evaluation of any NUS reconstruction. Most importantly, virtually all non-Fourier methods are nonlinear, and the nonlinearities manifest in different ways and to different extents. The same non-Fourier method may even produce varying degrees of nonlinearity when applied to data with different characteristics such as noise level, complexity, or dynamic range. An additional difficulty arises from how software packages may differently implement a similar theory or signal processing scheme. For example, results from algorithms that employ iterative soft thresholding (IST, [Bibr bib1.bibx12]), a class of CS methods that minimize the $\ell_{1}$ norm, differ depending on where the algorithm exits the fixed-point iteration. Algorithms that exit following a step in which samples in the time-domain instance of the reconstructed spectrum are replaced by the measured data yield reconstructions that exactly match the measured NUS data. This differs from algorithms that exit the iteration following the soft-thresholding step.

The protein-folding and molecular-docking communities have addressed similar obstacles in the evaluation of complex workflows with community-driven challenges. The Critical Assessment of Structure Prediction CASP challenged the protein-folding community by presenting amino acid sequences for recently solved (but unpublished) structures and then evaluating the structure submissions from contestants. This format helped the community develop and refine approaches based on template libraries, ab initio physical principles, and most recently machine learning. Similarly, the challenges of predicting how molecules bind has been addressed by the Critical Assessment of PRedicted Interactions CAPRI, resulting in numerous and powerful web-based docking platforms.

The Nonuniform Sampling Contest (NUScon) was conceived with inspiration from the CASP and CAPRI community initiatives. The objective of NUScon is to perform a critical assessment of NUS reconstruction tools, a task that required novel solutions to unique problems in designing the challenges and the scoring metrics to meet the needs of the broader NMR community. We met this objective by delivering a well-defined, workflow-based, quantitative platform that fosters access, development, and optimization of the wide range of NUS tools available to spectroscopists. This modular platform was designed to perform critical evaluations of each step in an NUS workflow with the goal of identifying best practices. NUScon provides the following deliverables: (1) a simple interface that improves access to the wide range of NUS sampling and reconstruction tools that are presently available but under-utilized; (2) a quantitative workflow and a corresponding series of NUS challenges designed to elucidate the best practices for collecting and processing NUS experiments; and (3) a public archive of challenge data, sample schedules, reconstruction scripts, spectra, and metrics that can be used as a benchmark when developing and optimizing new tools. The design of the synthetic data, the processing workflow, and the metrics will all evolve in response to knowledge gained from future rounds of NUScon. This continued refinement is especially true for the metrics as our community develops novel quantitative tools that capture essential qualitative spectral features. All of the resources presented here are distributed on the NMRbox computing platform [Bibr bib1.bibx28] and can be freely accessed by anyone with an academic, government, or non-profit affiliation.

## 2 Workflow

The objectives of NUScon were met by delivering a workflow-based software package that performs quantitative analyses of the quality of spectral reconstructions. A quantitative evaluation is dependent on how the “truth” is defined (i.e., the benchmark against which candidates are evaluated). While it may be tempting to use the DFT of a US experiment as the reference and then use an $\ell_{2}$ norm to compare it against a candidate NUS reconstruction, this approach fails on several counts. First, using the DFT as the reference is inappropriate since a goal of NUS is to improve spectral quality relative to DFT. Second, NUS reconstruction methods are nonlinear, so that metrics commonly used to quantitatively compare spectra obtained with the DFT are no longer appropriate. For example, SNR is not a reliable proxy for sensitivity. Similarly, root-mean-square (rms) differences may not be meaningful, although they often provide some insight.

In response to these obstacles, the NUScon workflow was designed around synthetic peaks. This ensures that the reference values (i.e., peak positions, intensities, and linewidths) are known quantities and that our metrics are based on their recovery. In addition, the synthetic peaks are injected into a uniformly sampled experimental data set, as opposed to being injected into a “clean” background or a background of experimental noise. This was a critical design choice as the CS results of [Bibr bib1.bibx11] show the dependence of all spectral reconstruction methods on the ratio between sampling coverage and signal sparsity. In other words, the recovery of synthetic peaks in the absence of experimental data (or noise) would present an artificial situation where the observed results are likely not applicable to real-world experiments. Another key benefit of using synthetic peaks is that an arbitrary number of peaks of varying characteristics may be injected. This ensures that spectral reconstruction workflows cannot be overfit to a given spectrum and thus remain transferable to similar experiments.

The NUScon workflow is illustrated in Fig. [Fig Ch1.F1], and the following subsections provide details about each component.

**Figure 1 Ch1.F1:**
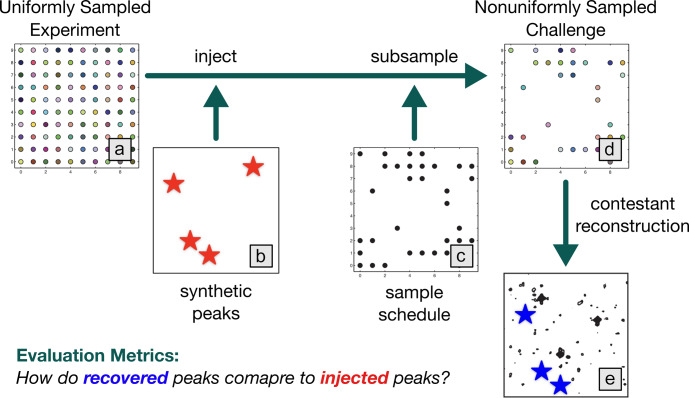
NUScon workflow. The uniformly sampled experiment **(a)** is injected with synthetic peaks (**b**, red stars) and subsampled **(c)** to generate a nonuniformly sampled challenge data set **(d)**. Contestants provide scripts to reconstruct the spectra **(e)**. The recovered peaks (**e**, blue stars) are compared against the injected peaks (**b**, red stars) using a variety of metrics to determine spectral quality.

### 2.1 Challenges

Each NUScon challenge began with an unpublished experiment that was collected with US. The Challenge Committee was tasked with identifying appropriate experimental data that are high quality and representative of molecular sizes and experiment types of interest to our community. The descriptions provided to the contestants for each molecule used in a challenge data set are given in Table [Table Ch1.T1]. A configuration file was deposited with each experiment, which provided basic metadata (e.g., number of points along the indirect dimensions, spectral width, and region of interest to be extracted along the direct dimension) that were used by the processing workflow. An example configuration file is included in the Supplement. We also include ${}^{1}$ H, ${}^{15}$ N projections from the 3D challenge data in the Supplement; these were not provided to contestants during the open challenge.

**Table 1 Ch1.T1:** Descriptions provided to the contestants for each of the molecules used in the challenge data.

Molecule tag	Description
proteinA	89 amino acid protein with residues 1–56 comprising a small well-folded protein fused with a linker and an IDP
proteinB	147 amino acid, 16 kDa protein that has a tendency to multimerize (monomer/dimer)
proteinC	107 amino acid protein, 12 kDa that is well-folded and well-behaved

### 2.2 Synthetic peaks

Evaluating the performance of a spectral reconstruction method is a challenging proposition, especially if the procedure must be automated. The central challenge is that NMR spectra are used for many purposes, and the properties that make a reconstruction most fit for purpose are not always the same. Also, in practice, parameters of interest are generally extracted from spectra rather than from time-domain data. So, while it is possible to make an unbiased, automated evaluation of agreement between observed time-domain data and the corresponding time-domain version of a reconstruction, this does not necessarily capture how effectively a reconstruction may serve a particular spectral analysis task.

Along these lines, while it may be possible to measure fidelity of frequency, amplitude, and signal envelope (decay) of individual signals, this does not necessarily capture the fitness of a reconstruction to suit a given use. In particular, while NUS reconstruction methods can have excellent lineshape fidelity [Bibr bib1.bibx50], it is seldom important in practice that a lineshape be conserved by a reconstruction, hence the common use of apodization that alters the lineshape, even if it conserves the integral [Bibr bib1.bibx37]. This is particularly true in multidimensional biomolecular data, where the indirect dimensions have little or no decay, so that the observed lineshape is primarily due to signal processing details rather than to any property of the underlying time-domain data. Furthermore, many applications, such as typical backbone assignment tasks, do not use peak heights or integrals quantitatively. Therefore, as a general point, it is often more useful to sharpen peaks or to suppress reconstruction artifacts than it is to faithfully reproduce lineshape.

The NUScon Planning Committee developed strong consensus that the single most important property of spectral reconstruction is for a real peak to be detectable, a fundamental requirement for all subsequent analyses. This, in turn, means that the evaluation of a reconstruction method is conflated with the procedure used to detect a peak. This is especially problematic for NMR, since the final decision about whether a spectral feature is a peak is often performed visually. Evaluation of the fidelity of the frequency, amplitude, and decay of a signal is likewise conflated with the procedure used to extract these parameters. The performance of such procedures is influenced by the presence and dynamic range of other peaks in the spectrum and by residual phase distortion, systematic artifacts, thermal noise, and the true functional form of the underlying NMR signals. Regarding this latter point, evaluations are further complicated by the potential for a single multidimensional peak to actually be a composite of mixed-phase signals in the presence of one or more unresolved couplings [Bibr bib1.bibx36] or the mixtures of signals with different relaxation pathways [Bibr bib1.bibx43].

Keeping these challenges in mind, we developed a flexible procedure to automatically define and insert synthetic signals into existing time-domain data. These synthetic signals have a similar appearance and arrangement to signals in the original data. For example, signals in an HNCA spectrum will generally occur in pairs at the same ${}^{1}$ H, ${}^{15}$ N chemical shift and will share the same lineshape in the ${}^{1}$ H and ${}^{15}$ N dimensions. Accordingly, the signal injection procedure provides options to specify how the synthetic signals should be placed relative to existing peaks. For example, we can specify that two synthetic HNCA peaks are inserted at a random ${}^{1}$ H, ${}^{15}$ N position where no peaks exist anywhere in the original 3D data but also that these two peaks are placed at ${}^{13}$ C positions that correspond to peaks elsewhere in the original data. In order to better mimic the appearance of measured data and to help minimize any evaluation bias due to use of synthetic data, the individual signals allow for random perturbations of linewidth and phase and optionally one or more random unresolved couplings in each dimension. It should be noted that there are opportunities to improve the realism of simulations still further, including using composite signals of mixed phases or linewidths or by attempting to match the coupling constants and decays of synthetic peaks to the values of related peaks in the measured spectrum. For example, in the current HNCA peak generation procedure, peaks can be inserted to align with a ${}^{13}$ C chemical shift of a peak elsewhere in the measured spectrum, and that synthetic peak will have randomly chosen ${}^{13}$ C decay and couplings. In a more realistic simulation, the synthetic peak could be generated to match not only the ${}^{13}$ C chemical shift of a peak elsewhere in the spectrum, but also that measured peak's ${}^{13}$ C decay and couplings, provided that these details are known.

The signal injection procedure automatically characterizes the experimental reference spectrum to prepare for the injection of synthetic peaks. The procedure was built from C-shell and TCL scripts using the facilities of NMRPipe and its custom TCL interpreter nmrWish [Bibr bib1.bibx10]. Here, we present an outline of an HNCA example used in the first NUScon, with complete data and scripts available on NMRbox [Bibr bib1.bibx28]. The input to the procedure was the measured reference 3D time-domain data, which were used to establish acquisition parameters used for simulating time-domain data, and the corresponding 3D spectrum, which was used to establish the phase values for simulated signals so that the simulated data can be processed by the exact same scheme applied to the measured data. The output of the procedure was a table of synthetic 3D signals, a 3D time-domain simulation with only the synthetic signals, and a version of the reference 3D time-domain data with the synthetic signals added.

1. The fully sampled 3D time-domain reference data are Fourier processed to generate a corresponding spectrum.
2. Automated peak detection (detection of local maxima) was applied, and the largest peaks were retained (256 peaks for this HNCA example). The NMRPipe peak detection procedure estimated heights and linewidths by a multidimensional parabolic fit of the points around the maxima.
3. Using knowledge of median linewidths from peak detection, each point in the 3D spectrum was evaluated to decide whether it was in an “empty” region, meaning that there are no substantial signals within ±2 median linewidths. The criteria are that the rms of the region must be below 1.15 times the estimated noise and that no points in the region are above 5.0 times the estimated noise. The results were saved as a mask of the 3D spectrum, where intensities were set to 1 for each point with an empty neighborhood and 0 otherwise. Two-dimensional projections of the 3D mask were also prepared as shown in Fig. [Fig Ch1.F2].
4. Using the peak and mask information from the steps above, the utility genSimTab.tcl was used to generate a synthetic peak table. Synthetic signals were only inserted in empty regions of the original data. Each row in the output peak table described one signal by a single amplitude and by a frequency, exponential decay, and phase for each dimension. Optionally, one or more couplings (cosine modulations) for each dimension may be specified. An example NMRPipe command illustrating the use of genSimTab.tcl is shown in Fig. [Fig Ch1.F3]. A key feature of genSimTab.tcl is that the desired properties of the signals to insert can be specified by a “program” of keywords and values, allowing substantial flexibility. Each program generates one or more “strips” (3D peaks at the same ${}^{1}$ H, ${}^{15}$ N coordinate). For HNCA data, each strip of synthetic signals consists of two 3D peaks. For example, the program
1 count=1,xy=novel,z=existing
  means generate one strip (count=1) of two peaks (HNCA peaks are generated in pairs) at a randomly chosen ${}^{1}$ H, ${}^{15}$ N location where no peaks exist anywhere in the original 3D data (xy=novel) but at ${}^{13}$ C positions of known peaks elsewhere in the spectrum (z=existing).
5. Synthetic time-domain data were generated by the simTimeND application using the synthetic peak table and the acquisition parameters from the measured data as inputs. This included the placement of a group delay of “negative time” points at the start of each 1D time-domain vector if the reference data did not properly account for digital oversampling, as is the case for data from Bruker spectrometers [Bibr bib1.bibx34]. The synthetic signals had exponential decays, with options for mixed-time data [Bibr bib1.bibx53]. The synthetic data also included a synthetic solvent signal of fixed width whose amplitude, phase, and frequency varied randomly in each 1D time-domain vector according to specified lower and upper bounds.
6. The synthetic time-domain data were added to the experimentally measured reference time-domain data. Example 2D projections and strip plots are shown in Figs. [Fig Ch1.F2] and [Fig Ch1.F4], respectively.
7. Given an appropriate sampling schedule, the fully sampled time-domain data can be resampled to generate NUS data via the nusCompress.tcl utility.

An advantage of this scheme is that it produces a spectrum processed identically to the experimental data but containing only synthetic peaks. This allows the simulated signals to be subjected to any desired detection or quantification method in either the presence or absence of signals and noise from the measured data.

**Figure 2 Ch1.F2:**
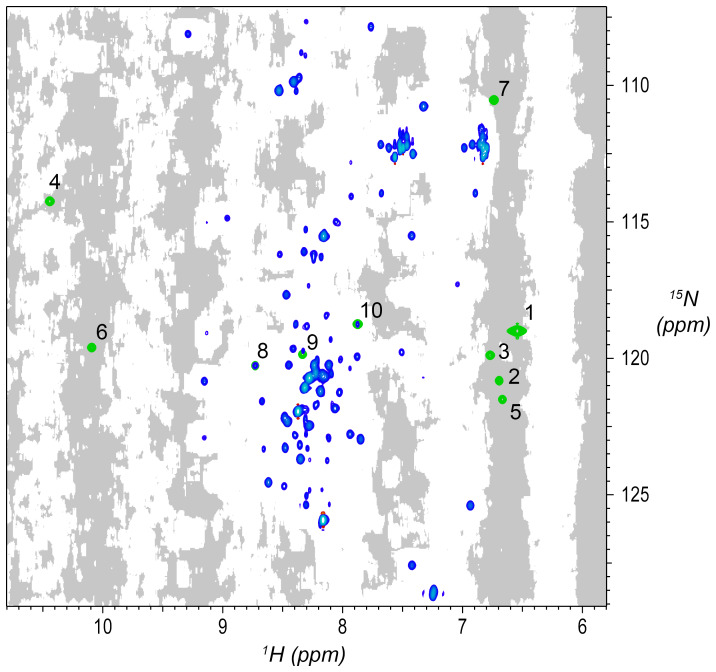
Synthetic peaks injected into HNCA empirical data. Two-dimensional projection of the 3D empty region mask (gray) overlayed with the 2D projections of the 3D reference spectrum (blue) and simulated signals (green). The areas shaded gray are regions in the measured data determined to be signal-free, such that a signal inserted at any shaded position will have no substantial signals nearby anywhere in the 3D spectrum. Synthetic signals were generated by the command in Fig. [Fig Ch1.F3], which generates 10 strips of synthetic peaks at the ${}^{1}$ H, ${}^{15}$ N positions labeled here. Strips from positions 7 to 10 are shown in Fig. [Fig Ch1.F4]. As can be seen here, strips 1 to 7 are at 2D coordinates with no existing peaks, strip 9 is at a 2D coordinate that partially overlaps with existing peaks, and strips 8 and 10 are at 2D coordinates that correspond exactly to existing peaks.

**Figure 3 Ch1.F3:**
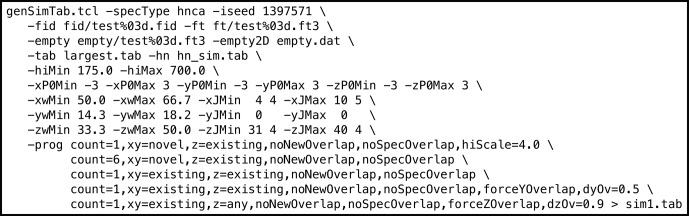
Example genSimTab.tcl command. This command generates a table of synthetic peaks for an HNCA spectrum.

**Figure 4 Ch1.F4:**
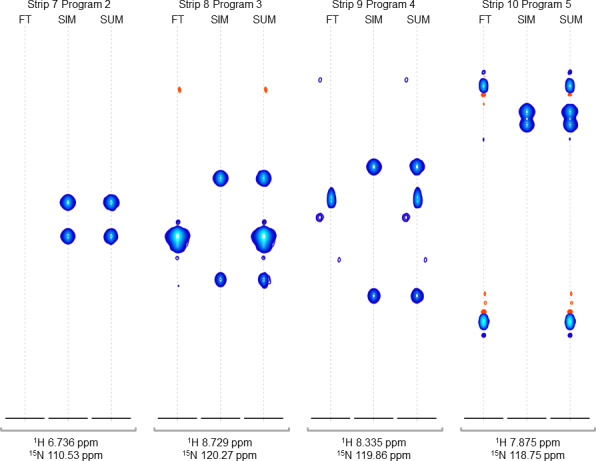
Strip plots of synthetic peaks. These strips show the auto-generated synthetic peaks and the corresponding measured data from the command in Fig. [Fig Ch1.F3]. The command uses five “programs” to produce strips of synthetic 3D signals at 10 ${}^{1}$ H, ${}^{15}$ N positions. The last four groups of strips (groups 7 to 10) are shown here, from the measured spectrum, simulated spectrum, and sum, labeled FT, SIM, and SUM, respectively. For these HNCA data, two simulated signals are inserted in every case. Strip group 7 corresponds to a program which inserts signals at ${}^{1}$ H, ${}^{15}$ N locations where there are no peaks anywhere in the original data, so that the “FT” strip in group 7 shows no signals. Strip group 8 corresponds to a program that inserts non-overlapping peaks at a ${}^{1}$ H, ${}^{15}$ N position where there are signals at other ${}^{13}$ C positions in the original data. Strip group 9 corresponds to a program that inserts peaks which are offset by 0.5 ppm in the ${}^{15}$ N dimension relative to an existing peak in the original data. Strip group 10 corresponds to a program which inserts two peaks that are 0.9 ppm apart in the ${}^{13}$ C dimension. It can also be seen that the measured data for this case have a combination of peaks with a broad appearance, as in FT strip 8, and peaks with a narrow appearance, as in FT strips 9 and 10.

### 2.3 NUS sample schedules

The NUScon software generates sample schedules by passing parameters to the nus-tool software package on NMRbox. The NUScon contest employed an exponentially biased sample schedule at a low and high coverage for each experiment. The parameters that defined each sample schedule are described in Table [Table Ch1.T2]. The public archive of NUScon data contains the sample schedules along with the complete nus-tool command used to generate each schedule, including the random seed.

Table [Table Ch1.T2] has a column that shows the “grid” size, which is the dimensions of the Nyquist grid that spans the indirect dimensions. Following the notation established in [Bibr bib1.bibx33], each point in a sampling grid is an indel (*indirect element*). The column in Table [Table Ch1.T2] labeled “Indels” shows the number of indels that were collected by the corresponding sample schedule. Each indel represents four FIDs for these three-dimensional experiments with quadrature detection employed. All schedules used “full component sampling,” so all four FIDs were collected at each indel. In future work, we may consider partial component sampling PCS;, where the group of FIDs at each indel may be subsampled.

**Table 2 Ch1.T2:** Challenge experiments and sample schedules. The left half of the table shows the NMR experiments that define each challenge data set. The right half of the table shows the sampling parameters used to generate a lower and a higher coverage exponentially biased sample schedule for each experiment. The column “Nuclei” lists the indirect dimensions, as specified in the fid.com scripts. The term “Indels” (i.e., “indirect elements”) is a single combination of evolution times taken across all indirect dimensions. Sample schedules customarily list the indels at which FIDs are collected. Dimensions that are constant time are indicated by zero-valued decay rates.

Experiment		Exponentially biased sample schedule				
Molecule	NMR	Nuclei	Grid	Indels (no.) (%)	Decay rate (Hz)	SW (Hz)
proteinA	HNCA	${}^{15}$ N, CA	90 × 44	125 (3.2 %)	15, 13	1563, 4951
				375 (9.5 %)		
proteinB	HNCA	${}^{15}$ N, ${}^{13}$ C	36 × 64	125 (5.4 %)	0, 102	1946, 4840
				375 (16.3 %)		
proteinA	HNCACB	${}^{15}$ N, CACB	90 × 95	250 (2.9 %)	23, 50	1563, 10870
				750 (8.8 %)		
proteinB	HNCACB	${}^{15}$ N, ${}^{13}$ C	32 × 64	200 (9.8 %)	0, 102	1622, 9434
				600 (29.3 %)		
proteinA	${}^{13}$ C NOESY-HSQC	${}^{13}$ C, H ${}_{\mathrm{ind}}$	93 × 150	1000 (7.2 %)	33, 55	3876, 6944
				3000 (21.5 %)		
proteinC	${}^{15}$ N NOESY-HSQC	${}^{1}$ H, ${}^{15}$ N	180 × 70	1500 (11.9 %)	63, 18	9300, 2500
				4500 (35.7 %)		

The nus-tool command to generate each sample schedule used input flags to force the collection of all FIDs at the “first” and “last” indels (i.e., the FIDs taken at the lowest and highest combinations of evolution times). The first indel, which contains the largest signal intensity, would very likely be sampled by any reasonable exponentially biased sampling schedule, but forcing its inclusion is an advantageous convention. The size of the Nyquist grid was explicitly defined in the NUScon workflow metadata. However, in practice, empirical data may not include such information, and the largest time increments observed in a sample schedule are often the best indicators of the size of the Nyquist grid. To comply with that convention, we forced the inclusion of the last indel.

### 2.4 Spectral reconstruction

We provided contestants with a template script for submission that accepts NUS time-domain data, a sample schedule, and various other parameters as inputs. Contestants were charged with using any of the tools on the NMRbox platform to process the data and produce a spectrum, subject to a basic set of rules. These rules include general guidelines, like the ethics statement “Any script that is not `reasonable' or tries to cheat the challenge problem is disqualified.” The rules also contain specific processing guidelines to ensure fair evaluations: “The spectrum produced by the user script must not exceed a final size larger than `rounding up' each dimension to the next Fourier number and 3 zero fills.” The rules document is available in the NUScon archive on NMRbox.

The range of reconstruction software programs employed is summarized in Table [Table Ch1.T3]. Several of the software packages provide access to various reconstruction algorithms, and some contestants used different algorithms for different challenges. The specific methods and parameters used by each contestant for each challenge should be referenced directly in their submission scripts inside the NUScon archive. It is worth noting that the SEER (from Hansen) and low-rank (from Qu and Lu) software packages were new to NMRbox and were provided along with the contest submissions. This showcases the ability of the NUScon workflow to promote the exploration of novel techniques and the agility of the NMRbox platform to readily incorporate them.

**Table 3 Ch1.T3:** Contestants and categorization of their chosen spectral reconstruction methods.

Contestant	Software	Algorithm class	Reference
Mark Bostock	CambridgeCS	IHT	[Bibr bib1.bibx6]
Frank Delaglio	NMRPipe	IST	[Bibr bib1.bibx10]
D. Flemming Hansen	SEER	Linear prediction	Unpublished work
Sven Hyberts	hmsIST	IST	[Bibr bib1.bibx19]
Bruce Johnson	NMRFx	NESTA	[Bibr bib1.bibx51]
Krzysztof Kazimierczuk	mddnmr	IST	[Bibr bib1.bibx24]
Hengfa Lu	XCloud-LR	Low rank	[Bibr bib1.bibx45]
Mark Maciejewski	RNMRTK	Maximum entropy	[Bibr bib1.bibx17]
Vladislav Orekhov	mddnmr	MDD and CS-IRLS	[Bibr bib1.bibx39]
Xiaobo Qu	XCloud-LR	Low rank	[Bibr bib1.bibx45]
Scott Robson	hmsIST	IST	[Bibr bib1.bibx19]
Jinfa Ying	NMRPipe/SMILE	Decomposition	[Bibr bib1.bibx54]
Matt Zambrello	RNMRTK	Maximum entropy	[Bibr bib1.bibx17]

### 2.5 Peak picking

The peak picking step in the workflow was simple to deliver but deceptively complex to interpret. We used the peak picker from NMRPipe, but setting the threshold and controlling the number of peaks recovered required some attention. The recovered peak table was used by the metrics discussed in the next section and, as will become clear, the number of peaks in the recovered table had an effect on the resulting metrics. As a consequence, the threshold used when peak picking had a direct effect on the metrics. Given that NUS reconstruction methods are nonlinear, there is no single intensity threshold that can be used across all reconstructions for a given data set. Therefore, the peak picking was performed with a low threshold; the resulting peak table was sorted by peak intensity and truncated to include 1500 of the most intense peaks. The fixed number was determined by manual inspection of the uniformly sampled empirical data and was set conservatively high to ensure that all true peaks would be captured.

The current approach based on a fixed number of recovered peaks was effective but will be replaced by the use of the in situ receiver operator characteristic IROC method in future rounds. The IROC method is advantageous since it continuously varies the peak picking threshold from the most intense peak down to the noise and reports the recovery rate and false discovery rate at each threshold. The resulting set of data defines the quality of the reconstruction independently of a specific peak picking threshold.

### 2.6 Metrics

The NUScon metrics were designed to quantify how well reconstruction workflows recover synthetically injected peaks. The metrics are briefly described here, and their full mathematical definitions are presented in Appendix A.

- M1: frequency accuracy. A symmetric Hausdorff metric was used with a maximum distance penalty to determine the accuracy of the recovered chemical shift positions. This metric allowed for different numbers of peaks in the injected and recovered sets (i.e., it does not require a one-to-one correspondence between the sets).
- M2: linearity of peak intensity. This quantifies how well the intensities of the recovered peaks were mapped by a linear function to their corresponding injected intensities. The correlation coefficient was calculated from the NumPy python package.
- M3: true positive rate. This reports the percentage of injected peaks that were recovered.
- M4: false positive rate. This reports the percentage of recovered peaks that were false.
- M5: linearity of peak intensity. This metric delivers the same evaluation as M2 but was implemented using an NMRPipe function.

### 2.7 Rank lists

A rank list was generated for each metric applied to each combination of experiment data, synthetic peak table, and NUS schedule. These rank lists are in the NUScon archive on NMRbox in comma-separated value (CSV) files. The report function in the NUScon software package can be used to extract arbitrary subsets of these rank lists to assemble aggregate results, which is how the NUScon challenges were evaluated.

## 3 Software

The NUScon software package is a modular, workflow-based, python3 software package that is installed on the NMRbox platform [Bibr bib1.bibx28]. NMRbox accounts are freely available to those with academic, government, and nonprofit affiliations. The NUScon software is licensed with GPL3. Users may run the software on NMRbox by executing the command nuscon, access the source code in /usr/software/nuscon, or import the NUScon python package into their own projects. NMRbox users can also execute the command nuscon -h to see a help message that defines input syntax and provides information about auto-generating a template file to run NUScon jobs. This publication uses NUScon software version 5.0.

The NUScon software was designed to manage a collection of project directories that contains the US experiment data, synthetic peak tables, sample schedules, and user submission scripts. As each evaluation was performed, workflow output was written to project directories that contain the spectra, projections, and peak tables. In addition, a JavaScript Object Notation (JSON) file was written to the output directory for each reconstruction that documented the metadata that defined the particular reconstruction, the compute times for each task in the NUScon workflow, and the metric scores. When rank lists were generated, the software simply queried the JSON files to aggregate all scoring data needed for the report. Compartmentalizing all of the workflow resources into nested project directories made it easy to run the NUScon workflow in parallel across the NMRbox computing cluster and to extract arbitrary subsets of evaluation data from the NUScon archive by experiment, user, metric, etc. JSON files are human-readable and contain a nested structure of key–value pairs. JSON files are easy for users to browse, efficient for computing workflows to parse, and straightforward to augment as new evaluation tasks may be retroactively performed on existing data.

It is worth noting that the design of the NUScon software made it trivial to deploy across the NMRbox cluster for parallel computing. Since each virtual machine (VM) in the NMRbox cluster has its own scratch disk, NUScon operations that were file input and output intensive saw tremendous performance improvements. All NMRbox VMs connect to the same file system, so the parallel jobs were written back into a common project. The NUScon software includes a status utility that reports on the progress of each evaluation task defined in the project.

The Supplement shows examples of the input configuration files and an output JSON file.

## 4 Results and discussion

A very generous gift from the Miriam and David Donoho Foundation allowed us to provide significant cash prizes to the contestants of the 2018 and 2019 challenges. This seed money provided incentive for the leading users and developers of the most prominent NUS software to participate, with the goal of ushering NUScon into a community-driven initiative that is self-sustaining. The publicly available software package is the first deliverable presented here, and the remainder of this section addresses the utilization of the software package to support two rounds of challenge problems.

Varying the type of experiment, nature of the synthetic peaks, sample schedule parameters (including coverage and random seed), and the metrics allowed for a comprehensive analysis of spectral reconstructions. It is important to note that while we customize the properties of the synthetic peaks for use with each metric, there are compounding factors to consider. When peaks are weak, broad, and/or close in proximity to additional peaks, they become difficult to recover. In particular, their sensitivity, frequency accuracy, and resolution are intimately connected, and quantitatively isolating any single property becomes challenging. We strive to minimize these complicating factors in our evaluation workflow by using a variety of synthetic peak collections for each experiment; the -prog section of the command in Fig. [Fig Ch1.F3] defines five different strips of synthetic peaks. Strong synthetic peaks in closely spaced pairs are employed to assess resolution, strong peaks in isolation are used to evaluate frequency accuracy, and variable intensity peaks in isolation are employed to measure sensitivity.

In the context of NUScon, it was desirable to combine the scoring results for multiple data sets in order to evaluate each of the challenges, as summarized in Table [Table Ch1.T4]. Since all metrics are normalized onto the interval [0,1] , it may be tempting to combine multiple metrics through averaging. However, the dynamic range of each metric is hard to pre-determine, and thus a mean score would be dominated by whichever metric had the largest range of values. Instead, we chose to rank order all submissions for each metric on each data set, converted these ranks into percentiles (i.e., a representation that is independent of the number of contestants), and then combined results by averaging the percentiles. This approach has the consequence that adding new evaluation scripts to the archive of existing challenges requires the re-scoring of the percentiles for all evaluations. Computationally this is insignificant, but it is worth noting that percentile rankings are dependent on their context, whereas the raw scores will remain unchanged and are a better choice for running iterative optimization.

**Table 4 Ch1.T4:** NUScon challenges 2018/2019. Each row group (separated by horizontal lines) shows a NUScon challenge and the parameters that define it: “Experiment” defines the molecule and NMR experiment providing the uniformly sampled time-domain data. “Synthetic table” gives the index value(s) of the five synthetic peak tables that are generated for each experiment (see the Workflow discussion for information about the design of each). “NUS coverage” indicates the usage of the “low” and “high” coverage sample schedules generated for each experiment, as defined in Table [Table Ch1.T2]. “Metric” lists the index value(s) of the metrics used to evaluate the reconstructions (see the Workflow discussion for information about each metric). The “Scores” column shows the top-performing contestants for the given challenge along with their average percentile taken across the evaluations of all parameter combinations that define the challenge. Note that not all contestants provided a submission for each challenge. The entry “*Multiple*” indicates a tie; complete results available in the NUScon archive.

Challenge		Parameters				Scores	
Index	Theme	Experiment	Synthetic table	NUS coverage	Metric	Contestant	%
2018.1	Overall	proteinA + HNCA	1, 2, 3, 4, 5​​​​​​​	low, high	1,3,5	Ying	82
		proteinA + HNCACB				Hyberts	73
		proteinB + HNCA				Hansen	66
		proteinB + HNCACB					
2018.2	Fidelity	proteinA + HNCA	1, 2, 3, 4, 5	low, high	1,5	Ying	78
		proteinA + HNCACB				Hyberts	74
		proteinB + HNCA				Hansen	67
		proteinB + HNCACB					
2018.3	Detection	proteinA + HNCA	1, 2, 3, 4, 5	low, high	3	Ying	90
		proteinA + HNCACB				Hyberts	71
		proteinB + HNCA				Hansen	64
		proteinB + HNCACB					
2018.4	Sensitivity	proteinA + HNCA	5	low	3	Ying	89
		proteinA + HNCACB				Maciejewski	74
		proteinB + HNCA				Hyberts	67
		proteinB + HNCACB					
2018.5	Resolution	proteinA + HNCA	1	high	1	Ying	80
		proteinA + HNCACB				Hyberts	75
		proteinB + HNCA				Delaglio	66
		proteinB + HNCACB					
2019.1	Overall	proteinA + ${}^{13}$ C NOESY-HSQC	1, 2, 3, 4, 5	low, high	1,3,5	Ying	61
		proteinC + ${}^{15}$ N NOESY-HSQC				Bostock	58
						Hansen	52
2019.2	Fidelity	proteinA + ${}^{13}$ C NOESY-HSQC	1, 2, 3, 4, 5	low, high	1,5	Ying	60
		proteinC + ${}^{15}$ N NOESY-HSQC				Bostock	58
						Hansen	53
2019.3	Detection	proteinA + ${}^{13}$ C NOESY-HSQC	1, 2, 3, 4, 5	low, high	3	Ying	64
		proteinC + ${}^{15}$ N NOESY-HSQC				Bostock	58
						Hansen	51
2019.4	Sensitivity	proteinA + ${}^{13}$ C NOESY-HSQC	5	low	3	Ying	75
		proteinC + ${}^{15}$ N NOESY-HSQC				*Multiple*	45
							
2019.5	Resolution	proteinA + ${}^{13}$ C NOESY-HSQC	1	high	1	Ying	75
		proteinC + ${}^{15}$ N NOESY-HSQC				Hansen	65
						Kazimierczuk	55

Another consequence of using rank order is that a difference in rank may result from either a small change in the underlying raw scores when many contestants are tightly grouped or from a large change in the underlying raw score when contestants are well-separated. The former may be insignificant, whereas the latter may indicate an important difference in performance. As an illustrative example, consider an alternative version of metric M1 that computes differences in peak positions in parts per million (ppm) rather than in Hertz. This approach resulted in small changes in the raw scores, which in many of the challenges resulted in a shuffling of the ranked results. This example suggests one be cautious not to over-interpret the ranked results in Table [Table Ch1.T4].

The parameter combinations that defined each NUScon challenge are given in Table [Table Ch1.T4]. The NUScon archive inside NMRbox at /NUScon/archive/report contains CSV files showing the rank lists by contestant for each metric on each data set. There are also CSV files showing the aggregated ranked results for each of the challenges shown in Table [Table Ch1.T4]. Note that not all contestants provided a submission for each challenge; the CSV files of rank lists should be consulted to determine participation in each challenge. It is also important to note that the “Scores” column in Table [Table Ch1.T4] should be cautiously interpreted; it should not be used as a way to select a best reconstruction algorithm for an arbitrary experiment, and it should not be used to eliminate a reconstruction method from consideration. The challenge experiments, synthetic peaks, contestant reconstruction scripts, and metrics represent a diverse collection of data that will guide the community towards best practices. However, the collection is still a slice through a very complex workspace. We intend to expand the scope of NUScon and to continually refine the evaluation metrics through future rounds of challenges. This follows the successful framework used in CASP [Bibr bib1.bibx26] and will help drive our evolving understanding of the challenges we face with NUS NMR.

The summary of computations performed for NUScon is provided in Table [Table Ch1.T5]. These data are provided as a qualitative way to describe the overall computational costs of the NUScon challenges. Direct comparison of compute times between various methods is beyond the current scope of the project and would depend on the optimization of each method to run multi-threaded operations. In addition, the NMRbox platform is a shared community resource with active users. The NUScon evaluations were performed in this dynamic environment where resource availability fluctuates.

**Table 5 Ch1.T5:** Summary of computations. The number of spectral reconstructions and the compute times they required. Most of the reconstruction methods are multi-threaded, and all computations are spread across 10 NMRbox VMs to yield a substantial reduction in the “wall time” for completing the evaluations.

Experiment		Evaluation		Compute time
Molecule	NMR	Contestants	Spectra	Time (CPU × h)
proteinA	HNCA	12	120	2229
proteinB	HNCA	12	120	1333
proteinA	HNCACB	12	120	13 674
proteinB	HNCACB	12	120	1383
proteinA	${}^{13}$ C NOESY-HSQC	6	60	501
proteinC	${}^{15}$ N NOESY-HSQC	6	60	172
TOTAL			600	19 292

It is important to note that M2 was not used in the NUScon evaluation; M5 was used instead. M4 will not be used until IROC methods are incorporated (discussed in Conclusions). The number of peaks recovered was held constant, and M4 was thus redundant to M3. Rather than reducing the set of metrics supported by the NUScon software, we instead intend to support an arbitrary variety of metrics and welcome contributions from the community.

To ensure that the metrics were robust, we performed visual inspection of spectra to validate that differences in metric performance corresponded to expected differences in spectral quality. This ensured that future efforts to optimize reconstruction methods can be based on maximizing metric performance. Figure [Fig Ch1.F5] shows a side-by-side comparison of the best and worst performers for each metric for the protein A/HNCA challenge data. Row 1 shows a spectral region with two synthetic peaks, which are indicated by the intersections of the labeled vertical and horizontal lines. Panel a shows a reconstruction that displays some distortion and inaccuracy in the recovered peak positions (M1 score = 0.01), whereas panel b shows an improved recovery of peak position (M1 score = 0.10). Row 2 shows a spectral region with two relatively weak synthetic peaks. Panel c shows a reconstruction that fails to recover one of the peaks (M3 score = 0.10), and panel d recovers both peaks (M3 score = 0.85). Row 3 shows a scatter plot of the recovered peak intensities versus their injected intensities. Panel e has a broader spread as well as a fairly intense peak that is not recovered (data point on the horizontal axis), resulting in a poor correlation (M5 score = 0.40). Panel f shows a reconstruction with a better linearity of intensities (M5 score = 0.99).

**Figure 5 Ch1.F5:**
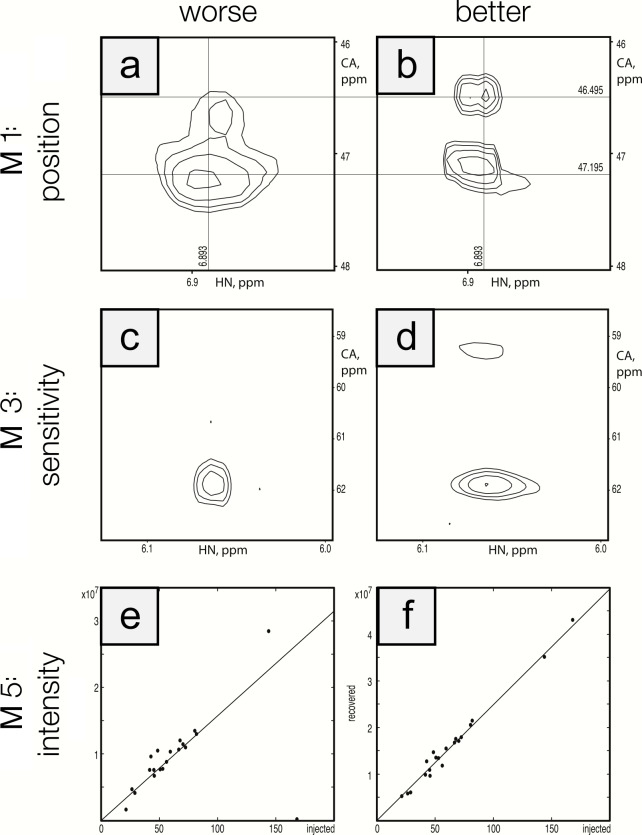
Visualization of metric performance. Reconstructions that perform relatively worse **(a, c, e)** and that perform relatively well **(b, d, f)** are shown for each of the metrics M1 (row 1), M3 (row 2), and M5 (row 3) on the protein A/HNCA challenge data. The crossing lines in panels **(a)** and **(b)** intersect where a pair of synthetic peaks has been injected. The peaks in panel **(a)** are clearly offset and distorted. Panels **(c)** and **(d)** show a pair of weak peaks, only one of which is recovered in panel **(c)**. Panels **(e)** and **(f)** show correlations between injected and recovered synthetic peak intensities.

## 5 Conclusions

The NUScon software, contest, and archive of spectral evaluation data provide a comprehensive platform for addressing the most challenging questions related to NUS experiments and even to broader topics in the quantitative analysis of NMR data. Establishing both a standardized workflow and a set of curated reference data holds tremendous power for the robust quantitative assessment of current methods and provides benchmarks by which novel methods may be compared and optimized. The NUScon workflow will expand in scope as new challenges are addressed (e.g., sample schedule design and peak picking), and the quantitative modules deployed in the workflow (e.g., metrics and peak simulation) will be subjected to continuous refinement.

A critical component of the NUScon workflow, which also has significant impact beyond the workflow presented here, is the genSimTab.tcl synthetic peak simulation package that Frank Delaglio delivered as an extension to NMRPipe [Bibr bib1.bibx10]. As discussed in the introduction and clearly presented by [Bibr bib1.bibx11], quantitative evaluations of spectral reconstructions require that candidate peaks (i.e., the synthetic peaks used here as a “ground truth”) be surrounded by other spectral data of suitable complexity and density. The synthetic peak simulation package was custom built to specifically deliver these needed features for the NMR experiments addressed in NUScon challenges. The package is extensible and can readily be maintained to support future challenges.

Peaks in a spectrum generated by conventional discrete Fourier processing will include truncation artifacts and the effects of any window function applied. This means that peaks in a discrete Fourier spectrum will never actually be simple ideal functions, such as Lorentzians, even though such simple functions can often be good approximations of observed lineshapes. In addition, measured NMR signals will deviate from ideal behavior in other ways, such as in the case when sample instabilities occur [Bibr bib1.bibx16]. The NUScon approach injects time-domain signals with known parameters into measured data and tests how well the injected signals can be recovered by the reconstruction method being evaluated. The synthetic time-domain signal injection approach, despite its advantages, comes at the price of a potential bias that was extensively discussed during the planning of NUScon. The synthetic signals are generated using ideal exponential decays, but several adjustments are applied in order to more effectively mimic experimental distortions. These adjustments include application of small random phase distortions, small random unresolved couplings, and addition of a synthetic solvent signal to induce baseline distortions. The simulated signals can also include placement of a group delay of “negative time” points at the start of each 1D time-domain vector to account for the potential distortion introduced by digital oversampling [Bibr bib1.bibx34]. Even with these attempts to generate more realistic simulations, since the signal injection procedure uses signals with ideal exponential decays as a starting point, this might still favor parametric reconstruction methods that use the same ideal model without conferring this benefit on non-parametric reconstruction methods.

The results captured in our archive of spectral reconstructions have shaped our preliminary definition of best practices and provide a quantitative benchmark. Future challenge problems (scheduled to be released annually in September/October, with results showcased at the Experimental NMR Conference in March/April) will enable us to build a database of quantitative evaluations across the entire NMR data processing workflow. We have thus far focused on NUS data reconstructions for common triple-resonance experiments used in protein NMR. We plan to expand the workflow by releasing challenges that address sample schedule design and peak picking. We plan to expand the scope of the contest to include data processing for experiments on small molecules, where the performance of the algorithms observed here for 3D protein NMR experiments may differ and require alternate optimization. We plan to offer challenges on 4D experiments where quantitative guidance in the description of best practices has great potential to significantly reduce experiment time and improve spectral quality for a class of powerful but under-utilized experiments. The reconstruction methods included in this work are among the most heavily used, but there are numerous other approaches. We plan to solicit submissions from developers, and we will initially focus on emerging tools in the area of machine learning. The modular design and open access to the NUScon platform provide an easy mechanism for integrating novel tools into the NUScon workflow, where they can be retroactively applied to all previous challenge data.

There are several features that are in development and scheduled for release during the next round of challenges. The first major planned development is the incorporation of the IROC method into the peak picking task; this will eliminate the need for an arbitrary peak picking threshold. We have written the IROC functionality into a stand-alone API and wrapped this using the Common Workflow Language CWL – a framework for defining analysis workflows.

The second major planned development is to provide the NUScon software with distributed computing capabilities. This is currently managed by manually running batches of evaluation tasks on various NMRbox virtual machines. However, NMRbox has an instance of HTCondor installed, and we will provide a mechanism to easily run distributed and balanced computing jobs on the NMRbox cluster. One possible approach is to wrap the entire NUScon workflow in CWL and make use of emerging high-throughput management systems like REANA, which has direct support for connecting CWL to HTCondor [Bibr bib1.bibx29].

The final major planned development is the implementation of a GUI front end, which will assist users with building NUScon evaluation jobs and browsing the database of quantitative results.

## Supplement

10.5194/mr-2-843-2021-supplement The supplement related to this article is available online at: https://doi.org/10.5194/mr-2-843-2021-supplement.

## Data Availability

The NUScon software package ( v5.0) is installed on NMRbox, and all contest data are archived and available on NMRbox at the mount point: .

## Dedication

We dedicate this publication to Nobel Laureate Richard R. Ernst for his extraordinary contributions to what we recognize as modern, high-resolution NMR. Ernst's development of pulsed NMR and subsequent usage of the Fourier transform improved the sensitivity of the technique by orders of magnitude, which opened up new applications across the biological and physical sciences. He revolutionized the field again with his contributions to the development of multidimensional experiments, which enhanced resolution and made the study of larger and more complex molecules possible. These monumental advances emerged in concert with the increasing power and availability of computational resources, leading Ernst to declare, “Without computers – no modern NMR” . The NUScon community continues in this spirit of discovery and gratefully acknowledges him.

## Appendix

The supplement related to this article is available online at: https://doi.org/10.5194/mr-2-843-2021-supplement.
