# Supplementary material for: NUScon: a community-driven platform for quantitative evaluation of nonuniform sampling in NMR
Source: Magn Reson (Gott). 2021 Nov 25;2(2):843–61. doi: 10.5194/mr-2-843-2021 (PMC10583271; doi:10.5194/mr-2-843-2021)
Supplement: The supplement related to this article is available online at: https://doi.org/10.5194/mr-2-843-2021-supplement. [file mr-2-843-supplement.pdf]

Supplement of Magn. Reson., 2, 843–861, 2021
https://doi.org/10.5194/mr-2-843-2021-supplement
© Author(s) 2021. CC BY 4.0 License.

Open Access
MAGNETIC RESONANCE

Supplement of

NUScon: a community-driven platform for quantitative evaluation of nonuniform sampling in NMR

Yulia Pustovalova et al.

Correspondence to: Adam D. Schuyler (schuyler@uchc.edu)

The copyright of individual parts of the supplement might differ from the article licence.

1 Software Inputs and Outputs

1.1 Input: master.cfg

The `master.cfg` file defines a structure for the complete project. The NUScon software includes a custom class based on the standard `configparser` python module that parses the path definitions in this file and dynamically substitutes parameters into the path definitions for each simulation as the resource is requested. This ensures that all project data for all simulations is handled consistently and it allows for simple augmentation if new files and resources are to be integrated into the NUScon evaluation. Copied below is a small fragment from the file and is meant to illustrate the simple human readable format and the variable substitution. Related parameters are grouped into sections with names in square brackets (*e.g.*, "`[path]`"). Lines within a section define key/value pairs. Values written as "`%%name%%`" are substitutions made at the time the resource is requested. Values written as "`${name}`" are cross-references to other values in the same section. Values written as "`${otherSection:name}`" are cross-references to values in other sections. Comma-separated lists are used to define nested directory paths.

```
[path]
experimentName = %%molecule%%_%%nmr%%
experiment = %%dirExperiment%%
    experiment_current = ${experiment}, ${experimentName}
        cfgFileExperiment = ${experiment_current}, experiment.cfg
        fid_us = ${experiment_current}, fid_us
            fid_data = ${fid_us}, fid
            proc_fid = ${fid_us}, fid.com
            proc_ft = ${fid_us}, nmr_ft.com
        fid_nus_synthetic = ${experiment_current}, fid_nus_synthetic
            fid_nus_current = ${fid_nus_synthetic}, synthetic-%%synthetic_index%%, ...
                ...schedule-%%schedule_index%%
                fid_nus_current_expand = ${fid_nus_current}, nus_expand, test%03d.fid
                fid_nus_current_fid = ${fid_nus_current}, nus.fid
                fid_nus_current_mask = ${fid_nus_current}, mask, test%03d.fid
```

For example, the time-domain data for a specific experiment is accessed with `getPath('fid_nus_current_fid')`.

1.2 Input: env.cfg

The `env.cfg` file defines environment variables that are defined for the shell that runs each reconstruction. Explicit definitions ensure that the NUScon environment is not subject to changes in the NMRbox platform and helps ensure that NUScon workflows are transferable and reproducible across the NMRbox cluster. Copied below is a small fragment from the file and is meant to illustrate how the file is structured and how it can easily be modified if needed.

```
[defs]
; define PATH and LD_LIBRARY_PATH as multi-line strings
; these are prepended to existing definitions
PATH =
    /usr/software/bin
    /usr/software/nmr-scripts
    /usr/local/sbin
    /usr/local/bin
    /usr/sbin
    /usr/bin
    /sbin
    /bin
    /snap/bin
    /usr/software/nmrpipe/nmrbin.linux212_64
    /usr/software/nmrpipe/com
    /usr/software/nmrpipe/dynamo/tcl

LD_LIBRARY_PATH =
    /usr/software/nmrpipe/nmrbin.linux212_64/lib

; define environment variables as key/value pairs
NVFXP = /NUScon/software/NMRFxProcessor/version-10.2.3/processor-10.2.3/nmrfxp
NMRPIPE_INIT = /usr/software/nmrpipe/com/nmrInit.linux212_64.com
PICKER = pkDetect3D.tcl
NUSTOOL = nus-tool-generate
```

1.3 Input: experiment.cfg

There is an `experiment.cfg` file embedded with each US experiment that documents some of the metadata for the experiment which is utilized by the NUScon workflow.

```
[proteinA_HNCA]
dim_names = 15N, CA
max_inc = 90, 44
SW = 1562.5, 4950.5
decay_rate = 23, 50
x1PPM = 10.3
xnPPM = 5.8
xP0 = -156.8
yP0 = 2.4

[metric]
# metrics look for a recovered peak within a half linewidth of the injected peak
# this is computed using the average LW (in Hz) of the injected peaks,
# as defined in the genSimTab script for the experiment
cutoff = 36.75

# the injected peaks are defined in PPM
# their position is needed in Hz for the metrics
# put the resonance frequencies defined in fid.com here (xOBS, yOBS, zOBS)
# (Hz = PPM * OBS)
OBS = 700.243, 70.963, 176.085
```

1.4 program.cfg

The `program.cfg` file defines where the project resources are located ("[project]" section), what NUScon tasks should be performed ("[run]" section), and what parameters should be used. The example below defines two jobs. The first job ("progWorkflowAll") generates sample schedules, injects synthetic peaks, performs reconstructions, peak picks spectra, computes scores, and gathers the scoring data. The second job ("doRank-2019.1") generates a ranked list of results for a specific NUScon Challenge by working with a subset of the data generated by the first job. In practice, the `program.cfg` file can be written with many jobs to either separate the workflow by task or to break the workflow into different sets of parameters so that jobs can be run in parallel. The `program.cfg` file has an extensive header that includes a full guide to running NUScon jobs.

```
[project]
dirSetting = /NUScon/archive/setting
dirExperiment = /NUScon/archive/experiment
dirSubmission = /NUScon/archive/submission
dirWorking = /scratch/%%user%%
dirSpectrum = %%HOME%%/myProject/spectrum
dirLog = %%HOME%%/myProject/log

[run]
programList = progWorkflowAll, doRank-2019.1

[progWorkflowAll]
task = sched, synth, recon, peak, score, gather
molecule = proteinA, proteinB, proteinC
nmr = HNCA, HNCACB, NOESYHSQC
synth_peak_index = 1,2,3,4,5
schedule_index = 1,3
user = contestant1, contestant2, contestant3
metric = 1,3,5

[doRank-2019.1]
task = rank
molecule = proteinA, proteinC
nmr = NOESYHSQC
synthetic_index = ${progWorkflowAll:synthetic_index}
schedule_index = ${progWorkflowAll:schedule_index}
user = ${progWorkflowAll:user}
metric = ${progWorkflowAll:metric}
rankFileBasename = challenge-2019.1-overall
```

1.5 Output: simData.json

This is a slightly simplified copy of an output json file where some long path names have been truncated to improve readability. Complete `simData.json` files are embedded with every NUScon evaluation output directory and are publicly accessible in the NUScon data archive. The "param" section documents the resource locations and current evaluation parameters. The "time" section records the wall, user, and system compute times for each task. The user compute time is multi-thread aware and reports on the total CPU time for the task. The "metric" section shows the raw scores for each computed metric.


```
{
    "param": {
        "dirExperiment": "/path/to/project/experiment",
        "dirSubmission": "/path/to/project/submission",
        "dirWorking": "/scratch",
        "dirLog": "/path/to/project/log",
        "dirSpectrum": "/path/to/project/spectrum",
        "dirRank": "/path/to/project/rank",
        "task": "recon",
        "molecule": "proteinA",
        "nmr": "HNCA",
        "synthetic_index": 1,
        "schedule_index": 1,
        "user": "contestant1"
    },
    "time": {
        "recon": {
            "wall": 247.5145070552826,
            "user": 1039.086282,
            "sys": 83.937606
        },
        "peak": {
            "wall": 378.1961841583252,
            "user": 370.63975500000026,
            "sys": 3.785168999999943
        },
        "score": {
            "m1": {
                "wall": 0.28973865509033203,
                "user": 0.248564,
                "sys": 0.008334000000000064
            },
            "m3": {
                "wall": 0.2306685447692871,
                "user": 0.22739200000000004,
                "sys": 0.003256000000003697
            },
            "m5": {
                "wall": 1.6490709781646729,
                "user": 1.198449,
                "sys": 0.017908999999999915
```

```
            }
        },
        "gather": {
            "wall": 0.0007526874542236328,
            "user": 0.00012200000000017752,
            "sys": 0.0001400000000000029
        }
    },
    "metric": {
        "m1": 0.5625487106185185,
        "m3": 0.95,
        "m5": 0.74681
    }
}
```

1.6 Synthetic Peak Simulation

Example `genSimTab.tcl` command for auto-generating a table of synthetic peaks for an HNCA spectrum; the command provides many options not shown here. Dimension-specific parameters are specified with usual NMRPipe nomenclature of X, Y, and Z for the three dimensions. Option `-specType` specifies the type of data being simulated, with the currently available types: `hnco hnca cbcaconh cbcanh n15Noe c13noe`. Type `hnca` means that peaks will be inserted in pairs at the same ^{1}H,^{15}N chemical shift, and will share the same lineshape in the ^{1}H and ^{15}N dimensions. The option `-iseed` specifies a random number seed. Options `-fid` and `-ft` specify the reference time-domain input data and corresponding input spectrum; these are used to determine the acquisition parameters and phases that should be used to generate the synthetic data. Option `-empty` specifies the 3D spectrum mask input that identifies signal-free regions in the reference spectrum, while option `-empty2D` specifies the 2D mask that identifies empty regions in the XY (here, ^{1}H,^{15}N) plane (Fig. **??**). Option `-tab` specifies the input peak table of the largest peaks in the reference spectrum, and option `-hn` specifies an output table that will contain the ^{1}H,^{15}N coordinates where one or more synthetic peaks were generated; this table can be used later for viewing strips of the simulated signals. Several simulation parameters are Gauss-random values clipped to fall within specified upper and lower bounds, using a Gaussian distribution whose standard deviation is 0.35 times the range of the bounds. Options `-hiMin -hiMax` specify the range of Gauss-random time-domain amplitudes for the signals generated. Options `-xP0Min -xP0Max` etc. specify the ranges of Gauss-random phase perturbations in degrees to add in each dimension. Options `-xwMin -xwMax` etc. specify the ranges of Gauss-random time-domain decays in each dimension, in Hz. Options `-xJMin -xJMax` etc. specify the lower and upper bounds in Hz for one or more Gauss-random cosine modulations in each dimension, in Hz. The `-prog` option specifies details for one or more "strips" (related peaks at the same ^{1}H,^{15}N coordinate) of synthetic peaks to create. Details are specified as one or more "programs", where each program is comma-separated list of keywords and values that describe the desired properties of the synthetic signals, including how their positions should be chosen. The program scheme provides substantial flexibility to generate synthetic signals, although some combinations of settings are redundant, and it is possible to specify contradictory combinations of options that can't be achieved. In this example, there are five programs generating a total of 10 strips:

1. `count=1,xy=novel,z=existing,noNewOverlap,noSpecOverlap,hiScale=4.0`
   Generate a single strip (`count=1`) of 3D peaks. `xy=novel` means the peak will be inserted at an empty ^{1}H,^{15}N location (*i.e.*, no existing peaks at that ^{1}H,^{15}N location anywhere in the 3D spectrum). `z=existing` means the peaks will have ^{13}C positions that correspond with peaks elsewhere in the spectrum, according to table of largest peaks in the measured spectrum (largest.tab). Note that while these synthetic peaks will have ^{13}C chemical shifts that match existing peaks in the spectrum, the ^{13}C lineshapes of the synthetic peaks are independent of existing peaks. Keyword `noNewOverlap` means the simulated peaks will not overlap other simulated peaks. Keyword `noSpecOverlap` means the simulated peaks will not overlap known peaks in the measured spectrum. Keyword/value `hiScale=4.0` means the peak intensities will be scaled up by a factor of 4.

2. `count=6,xy=novel,z=existing,noNewOverlap,noSpecOverlap`
   Generate six strips (`count=6`) with the default height range, using the same parameters as program 1.

3. `count=1,xy=existing,z=existing,noNewOverlap,noSpecOverlap`
   Generate one strip at the same ^{1}H,^{15}N coordinate as an existing peak in the measured data (`xy=existing`), although the ^{15}N lineshapes of the synthetic signal are independent of existing peaks. As before, `z=existing` means the peaks will have ^{13}C positions that correspond with peaks elsewhere in the spectrum.

4. `count=1,xy=existing,z=existing,noNewOverlap,forceYOverlap,dyOv=0.5`
   Generate one strip according to the ^{1}H,^{15}N coordinate of an existing peak in the measured data (`xy=existing`), but displace the peaks by 0.5 ppm in the ^{15}N dimension (`forceYOverlap,dyOv=0.5`).

5. `count=1,xy=existing,z=any,noNewOverlap,noSpecOverlap,forceZOverlap,dzOv=0.9`
   Generate one strip with a pair of peaks that overlap by 0.9 ppm in the ^{13}C dimension (`forceZOverlap,dzOv=0.9`).

2 NMR Projections

The uniformly collected, empirical data for each protein is Fourier Transformed with an NMRPipe auto-generated processing script (nmr_ft.com), which is available in the NUScon archive on NMRbox. The resulting 3D spectra are projected onto the 1H and 15N axes and shown in Figure S1. These projections were not provided to the contestants during the open challenge, but are presented here for reference. The contestants were provided information about the size of each challenge protein used in the contest, as discussed in the manuscript.

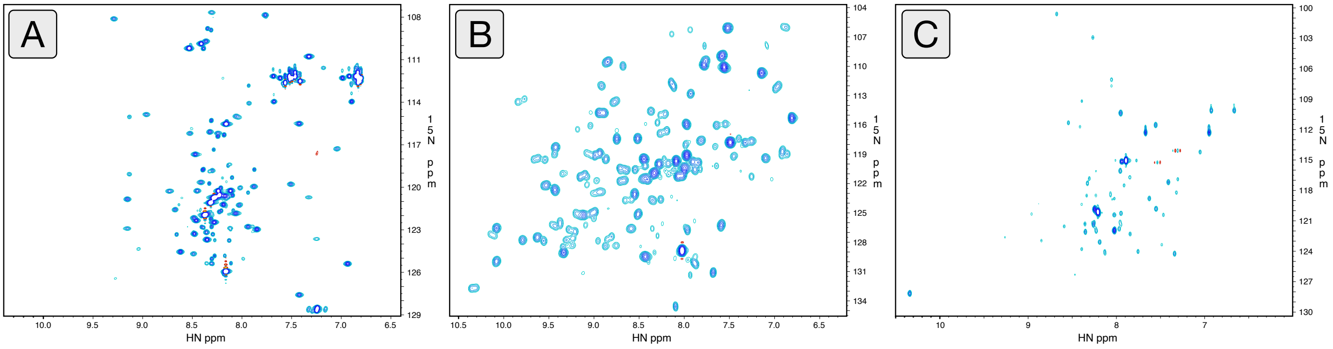

Figure S1. 1H-15N Projections for each Challenge Protein. Projections are shown for proteinA / HNCA (**A**), proteinB / HNCA (**B**), and proteinC / NOESY-HSQC (**C**).
